# Quantifying and predicting depression literacy of undergraduates: a cross sectional study in Sri Lanka

**DOI:** 10.1186/s12888-015-0658-8

**Published:** 2015-10-30

**Authors:** Santushi D. Amarasuriya, Anthony F. Jorm, Nicola J. Reavley

**Affiliations:** Behavioural Sciences Stream, Faculty of Medicine, University of Colombo, 25, Kynsey Road, PO Box 271, Colombo 8 Colombo, Sri Lanka; Centre for Mental Health, Melbourne School of Population and Global Health, University of Melbourne, 207 Bouverie Street, Parkville, Victoria 3010 Melbourne, Australia

**Keywords:** Depression literacy, Mental health literacy, Depression, Undergraduate, Recognition, Treatment beliefs, Scale development, Help-seeking

## Abstract

**Background:**

The high rates of depression and low rates of related help-seeking among undergraduates are matters for concern. In response to the need to examine their knowledge about depression and its management, and the dearth of such research from non-western developing countries, this study examined the depression literacy of undergraduates in Sri Lanka.

**Methods:**

A questionnaire was administered among 4671 undergraduates to examine their depression literacy relating to problem-recognition, measured using a vignette of a depressed undergraduate, and their treatment beliefs measured by assessing their perceptions about the helpfulness of various options of help for the presented problem. Responses for the latter aspect were quantified using a scale comprising the options of help endorsed by Sri Lankan mental health professionals. Regression analysis models were used to identify the correlates of these aspects of depression literacy.

**Results:**

Females, medical undergraduates and those in higher years of study (compared to first-years) were more likely to recognise the problem as depression. The undergraduates obtained a mean percentage score of 76 % on the constructed Depression Treatment Beliefs Scale. Scores on this scale were higher among females, medical undergraduates, those who got help for the problem after trying to deal with it alone and those who recognised the problem as depression, as well as those who used other mental health-related labels for this purpose. Scores were lower among undergraduates in years 2–4 (compared to first-years), those with family or friends with the problem and those with higher stigma on a Social Distance Scale. However, the effect sizes of these relationships were small.

**Conclusions:**

As factors such as gender, discipline, year of study, exposure to depression and stigma are associated with differences in the depression literacy of these undergraduates, concerning their ability to recognise the problem and their related treatment beliefs, these must be considered when designing related educational initiatives. Recognising the problem as depression or the use of other mental health-related labels is associated with better treatment beliefs as per expert consensus, indicating that such labelling could have value for appropriate help-seeking.

**Electronic supplementary material:**

The online version of this article (doi:10.1186/s12888-015-0658-8) contains supplementary material, which is available to authorized users.

## Background

‘Mental health literacy’ is described as the possession of knowledge and beliefs which facilitate the recognition, management and prevention of mental disorders [1]. The high rates of depression among undergraduates [2, 3], their low rates of mental health related help-seeking [4–6], and evidence that a young person’s mental health literacy is associated with their help-seeking practices [7, 8], highlight the need for examining and improving the *depression literacy* of undergraduates.

Unfortunately, a majority of mental health literacy studies and more specifically, those concerning the depression literacy of undergraduates have been in developed western countries, resulting in limited understanding of this area in developing and non-western countries [9, 10]. This gap in research must be urgently addressed, as the depression literacy of undergraduates in these contexts might differ from those from a western cultural background [11–13]. Furthermore, many developing countries have a scarcity of mental health resources [10] and this might influence the depression-related knowledge and responses of undergraduates in these countries. Hence, their depression literacy must be examined whilst also taking into account their unique socio-cultural context. Furthermore, these studies must identify those with depression literacy deficits to guide related interventions for undergraduates, which could be carried out as a part of whole-of-community campaigns or through educational programmes, mental health first aid training and information websites [14].

The present study focused on examining the depression literacy of undergraduates in Sri Lanka. Previous studies have identified a range of culturally-sanctioned mental health responses and beliefs of the Sri Lankan population, such as traditional modes of healing [15–19]. However, research also indicates that these exist alongside the population’s use and endorsement of professional help, such as from psychiatrists and doctors [16, 18]. While there have been only two prior mental health literacy surveys in Sri Lanka, one being on carers of the mentally ill [18] and the other on health professionals and the general public [20], they provide evidence for the aforementioned trend [18], as well as for this population’s endorsement of their social network, such as family [18, 20]. However, the informal options of help might be easier to engage in and more accessible than mental health professionals, who are in short supply [21]. Hence, it becomes necessary to understand this population’s knowledge about treatment options for mental illness, given the cultural milieu within which they exist. The absence of such mental health literacy research on undergraduates highlights the need for the present study.

The need for this examination is emphasised by the high rates of psychological distress and depression symptomatology found among undergraduates in Sri Lanka [22, 23]. Amarasuriya et al. [24] found that close to 10 % of undergraduates at the University of Colombo, in Sri Lanka screened positive for Major Depressive Disorder. The importance of assessing the depression literacy of this group is also indicated by evidence of their stigmatising attitudes towards peers with depression (e.g. that the symptoms are due to a *weakness* and not a *sickness*) [25] and findings indicating that such stigma might influence their beliefs about help-seeking [26].

### Measurement of disorder-related literacy

Studies examining the mental health literacy of populations have focussed on their recognition of and treatment beliefs about disorders, and have typically assessed these aspects by using the related opinions of health professionals as a benchmark [27, 28]. The benefits of using such a strategy to assess the treatment beliefs of a population are two-fold. These *experts’* opinions could be considered to reflect, at least to some extent, the treatment practices that are routinely prescribed by health professionals. Furthermore, when these experts are from the same context as the population being assessed, their opinions could be considered to also reflect culturally relevant strategies for dealing with the problem and hence, provide a contextually sensitive benchmark for assessing mental health literacy.

In assessing the mental health literacy of the general Australian community, Reavley et al. [29] developed a scale that assessed their beliefs about the helpfulness of a range of treatment options endorsed by health professionals. This enabled the generation of a scale score reflecting the degree to which the target population agreed with the treatment beliefs of professionals. This scale incorporated not only professional strategies but also informal and self-help strategies for dealing with the examined mental disorders, as per expert consensus. This methodology could be especially useful for assessing mental health literacy in developing countries such as Sri Lanka, where the population might utilise a wide array of help-seeking options for their problems. While this method enables an evaluation of which of these are recommended by professionals, it also enables an assessment of whether the population is knowledgeable about these recommended options of help. As noted earlier, it is also necessary to examine the factors associated with such depression literacy in order to identify those who need to be targeted in depression literacy initiatives. Previous mental health literacy studies that have quantified participants’ responses have found that factors such as gender, age, education, exposure to mental illness and stigma are associated with differences in the generated scores [27, 29].

The present study aimed to examine the depression literacy of undergraduates in Sri Lanka, with regard to their ability to recognise the disorder and their related treatment beliefs, and to identify the correlates of such depression literacy, focussing on their demographic characteristics, exposure to depression and attitudes about those affected by it. In line with Reavley et al. [29], treatment beliefs were quantified by using a scale consisting of the options of help that were endorsed by Sri Lankan mental health professionals.

## Methods

### Design, participants and setting

This cross-sectional study was conducted from June to November 2013 among undergraduates at the University of Colombo, one of the largest universities in Sri Lanka [30]. The present paper, the Amarasuriya et al. [24] paper examining depression among undergraduates in Sri Lanka and the Amarasuriya et al. [25] paper examining stigma among this population, were all based on the same Depression Literacy Survey conducted among this undergraduate population at the University of Colombo.

The research sites included five of the six undergraduate faculties of the university, namely, the faculties of Arts, Law, Management and Finance, Medicine, Science and the School of Computing, an affiliated institute of the University. The sampling strategy aimed to produce as large a sample as possible, by approaching all those who attended a lecture identified as being common in each year of study in the research sites. We attempted to reduce any bias by systematically approaching undergraduates from all faculties/schools during lectures. In the case of the Faculty of Arts where undergraduates had varied subject combinations, undergraduates who attended lectures with the largest student cohorts were approached. Data was not collected from the Faculty of Education as it was expected that the second and third year students of this faculty would be approached at the lectures they attend at the Faculty of Arts and as only the fourth year students had lectures exclusively at this faculty.

### Measures

#### Cultural adaptation of measure

Mental health literacy surveys used among the adult population [1] as well as undergraduates [31] provided the basic template for developing the questionnaire used in the Depression Literacy Survey. The questionnaire underwent several stages of adaptation, including incorporation of items relevant to the target population and the broader Sri Lankan mental health context as seen in prior research. Mental health literacy surveys previously used in Sri Lanka were also reviewed within this process [18, 20]. Subsequent to this, the measure was reviewed for cultural relevance first by Sri Lankan postgraduates at the University of Melbourne who had completed their undergraduate studies in Sri Lanka (three groups of 4–6 members), and then by mental health professionals in Sri Lanka (*n* = 7). The adapted questionnaire was then translated from English, into Sinhala and Tamil by two professional translators. The questionnaire was in two versions, as either English-Sinhala or English-Tamil, with both versions containing the questions in English and participants able to use the version with their preferred translation. The English-Sinhala version was checked for translation accuracy by a clinical psychologist, senior registrar and registrar in psychiatry, and the English-Tamil version was checked by a clinical psychologist, all conversant in the relevant translation languages. The questionnaire was piloted among ten undergraduates at the University of Colombo prior to finalisation. Please see Additional file 1 for the English-Sinhala version of the questionnaire.

#### Variables measured

Subsequent to a section for demographic information (gender, age, faculty, year of study, residence, religion, ethnicity, district), a vignette was presented of an undergraduate named “Z”, exhibiting symptoms of Major Depression as per the Diagnostic and Statistical Manual of Mental Disorders-IV [32] (Please see Additional file 1 for vignette). Participants were instructed to consider “Z” to be of their same age and gender.

Depression literacy relating to problem-recognition was examined using an open-ended question which asked participants what they thought was wrong with “Z”. Two additional questions examined their help-seeking intentions and mental health first-aid responses towards “Z” (not examined in the present paper).

As in similar studies among undergraduates [31, 33–35], the participants’ treatment beliefs were examined in relation to their perceptions about the helpfulness of a range of help-providers and interventions to assist “Z” to deal with the problem (rated as ‘very helpful’, ‘fairly helpful’, ‘neither helpful nor unhelpful’, ‘fairly unhelpful’, ‘very unhelpful’, ‘don’t know’). The Depression Literacy Survey questionnaire consisted of a total of 50 such items subsequent to cultural adaptation (please see Additional file 1 for these items).

As mentioned earlier, the undergraduates’ stigmatising attitudes and exposure to mental illness were also examined, given previous findings that these factors are associated with mental health literacy. Stigmatising attitudes were examined using scales assessing participants’ personal stigma towards “Z” (Personal Stigma Scale) [36, 37] and their willingness to have social contact with “Z” (Social Distance Scale) [37, 38]. The participants’ exposure to depression was examined using questions about whether anyone in their family or close circle of friends had a problem like “Z” (response options: Yes, No, Don’t know) and if they ever had a problem like “Z” (response options: Yes, No, Don’t know). In the case of the latter, participants were asked if they dealt with the problem on their own, without getting help from others (response options: Yes, Tried first but got help later, No). The questionnaire also examined if participants were personally experiencing depression symptomatology through the use of the Patient Health Questionnaire-9 (PHQ-9) and its Sinhala or Tamil adapted versions [20] . This was done in relation to whether they screened positive for Major Depression (diagnosis given if five or more of the PHQ-9 symptoms were present at least “more than half the days” in the past two weeks, with the symptoms of either depressed mood or anhedonia present. If the symptom on suicidal thoughts was present at all, it was considered in the symptom count for the diagnosis [39]).

### Procedure

The paper-based questionnaire was administered during lectures among undergraduates in all years of study at the relevant faculties/institutes. During distribution of the questionnaires, the potential participants were given a brief introduction to the study, mostly by SDA or, in her absence, by the relevant lecturer who read out an introductory statement. The undergraduates were also informed that participation was voluntary. They were then referred to the participant information sheet. The participants took approximately 20 min to complete the questionnaire.

#### Examination of problem-recognition

##### Coding of responses for problem-recognition question

Coding of responses to this question (asking participants what they thought was wrong with “Z”) was done by SDA, a clinical psychologist trained in Sri Lanka, who is fluent in Sinhala and English, the languages used by most participants. SDA coded the English translations of the Tamil responses which were provided by a professional translator. Pre-coded categories used in similar research were used as a guide when coding the responses [1, 40]. However, as similar work had not been done previously among this undergraduate population, coding categories were created for all responses which varied in meaning. Each of the categories obtained for this question was coded as ‘yes’ or ‘no’ where multiple categories could be coded (e.g. *mental problem, mental unrest, mentally in a mess, mental break down*). Subsequent to this, the authors categorised similar codes into broader coding categories, with the final categories being those nominated by ≥ 5 % (e.g. the aforementioned codes were categorised into the broader coding category ‘mental issue’). If a coding category was nominated by ≥ 2 % to ≤ 5 % respondents, but was distinct and approximated correct recognition of the condition, such categories were also permitted to constitute a final coding category. The final seven coding categories were, ‘depression’, ‘mental illness’, ‘mental issue’, ‘stress, pressure, mental suffering’, ‘university/education related problems’, ‘romantic relationship related problems’, with all other responses assigned to an ‘other’ category (see Amarasuriya et al. [41] for more details regarding the coding categories).

The present paper examined problem-recognition in relation to the undergraduates’ ability to recognise ‘depression’. Only 17.4 % of the study sample recognised the condition [41]. However, as 53.8 % of respondents recognised the condition using a range of other mental health-related labels (relevant to the coding categories ‘mental illness’, mental issue’ and ‘stress, pressure, mental suffering’) problem-recognition was also examined in relation to the use of such labels.

Although the scale constructed by Reavley et al. [29] assessed both the participants’ ability to correctly recognise the problem in the questionnaire vignette as *depression* (1 point awarded) and their treatment beliefs, leading to the generation of an overall score, we examined these two aspects of depression literacy separately given the low rate of recognition of depression.

#### Examination of treatment beliefs

##### Development of depression treatment beliefs scale

An online survey listing the 50 help-providers and interventions for dealing with depression, which were rated by the undergraduates, was administered among Sri Lankan psychiatrists and clinical psychologists. A total of 37 valid responses were obtained (psychiatrists = 21; clinical psychologists = 12; not specified = 4). The response rate was 36 % of the total population of these mental health professionals identified via their respective professional/registration bodies. Items for which there was consensus among ≥ 75 % of the *mental health experts* that these were ‘helpful’ (either ‘very helpful’ or ‘fairly helpful’) or ‘unhelpful’ (either ‘very unhelpful’ or ‘fairly unhelpful’) when dealing with depression, were included in the constructed Depression Treatment Beliefs Scale which consisted a total of 23 items.

Items endorsed as ‘helpful’ were: a psychiatrist; a psychologist; a counsellor; an organisation helping people to deal with problems; a university student counsellor; a university medical officer; a mental health professional at the university psychiatry unit; parents; boyfriend/girlfriend/spouse; a friend from university; get counselling or psychological therapy; take western medicine to improve mood; become more active in daily activities; do physical exercise; do activities he/she enjoys; do meditation, yoga or other relaxation exercises; improve sleeping habits; get information from the internet about dealing with problem; talk to others who have faced similar problems; cut down use of alcohol/cigarettes/drugs.

Items rated as ‘unhelpful’ were: not approach anyone for help and deal with problem alone; stop going to university and stay at home; use alcohol/cigarettes/drugs.

##### Scoring of undergraduate responses

As described in Reavley et al. [29], 1 point was awarded for each item that the undergraduates rated as ‘helpful’ (either ‘very helpful’ or ‘fairly helpful’) that had been classified as such by the mental health experts (total of 20 points) and, similarly, 1 point each was awarded for items that they rated as ‘unhelpful’ (either ‘very unhelpful’ or ‘fairly unhelpful’), that had been classified as such by the experts (total of 3 points). This resulted in a maximum scale score of 23.

Therefore, the scores that the undergraduates obtained on this 23-item Depression Treatment Beliefs Scale indicate the degree to which they agreed with experts about treatments and options of help for depression, with higher scores indicating greater agreement. The undergraduates’ depression literacy, with regard to their treatment beliefs, is considered in relation to the scores that they obtained on this scale in the present paper.

### Ethics approval

Approval for this study, including for administering the online survey among the mental health professionals, was obtained from the Ethics Review Committees of the Faculty of Medicine, University of Colombo, and University of Melbourne.

The participant information sheet that was presented to undergraduates along with the study measure provided details about the study, including that if a filled questionnaire was returned that this implied the respondent’s consent to participate in the study. Such a passive consent approach was considered to be appropriate as the identity of participants remained anonymous.

### Statistical analysis

The internal consistency estimates (Cronbach’s alpha and McDonald’s Omega) and descriptive statistics for the Depression Treatment Beliefs Scale were found. 10 % of missing items were permitted (two items) with the missing values prorated using the mean of the existing item-ratings.

Regression analysis models were used to examine if the undergraduates’ depression literacy (problem recognised as depression or by using other mental health-related labels and scores on the Depression Treatment Beliefs Scale- DVs) were predicted by participants’ demographic characteristics and their exposure to and attitudes about depression, as seen in previous research [27, 29]. Univariate regression analysis models were used to examine the association that each of the predictor variables (IVs) had with the depression literacy measures (DVs). Multiple regression analysis models were also used, where all IVs were entered into a single model simultaneously to examine the associations that each of the IVs had with the DVs while simultaneously adjusting for the effects of the other variables. As there were a large number of predictors examined the *p* < .01 level of significance was used to reduce the Type I error rate.

Accordingly, multinomial logistic regression was used to examine the predictors for recognising the problem in the vignette as either depression or by using other mental health-related labels (DVs); the reference group being those not using such labels. When responses for recognition were relevant to both the *depression* and *other mental health-related problem* label categories (e.g., “the problem is either stress or depression”), these were only coded for the response category indicating recognition of ‘depression’. The following variables were examined as predictors (IVs) of problem-recognition: gender, faculty of study, year of study, age category, residence, religion and the presence of Major Depression as per the PHQ-9.

A linear regression model was used to examine the predictors of the scale scores (DV). The following dummy coded variables (IVs) were examined as predictors: gender, faculty of study, year of study, age category, residence, religion, presence of Major Depression as per the PHQ-9, if respondents had a family member or friend who experienced the problem, if they had personally experienced the problem and if so, whether help was sought (with those not indicating personal experience of the problem included in the analysis but dummy coded as a ‘not relevant’ category) and ability to recognise the problem as depression or by using other mental health-related labels. Scores obtained on the Personal Stigma and Social Distance Scales (continuous variables) were also examined as predictors of the scale scores. Amarasuriya et al. [25] found that the Personal Stigma Scale consisted of two dimensions of stigma (i.e., the “Weak-not-Sick” and “Dangerous- Undesirable” dimensions), and that the Social Distance Scale consisted of one dimension (i.e., the “Social Distance” dimension). Hence, the participants’ stigma scores on these measures were entered into the model in relation to the Weak-not-Sick, Dangerous-Undesirable and Social Distance scales that were constructed in relation to the identified dimensions of stigma (the latter scale being the same as the original social distance measure) [25].

## Results

Almost all undergraduates who were approached for the survey participated, with a total of 4671 valid responses (response rate approaching 100 %). This was approximately 52 % of the undergraduates at the University of Colombo. Table 1 presents the demographic and other relevant characteristics of the respondents. Descriptive data on the stigma scales have been previously reported [25].

**Table 1:** Demographic and other characteristics of the undergraduate sample (*n* = 4671)

Variables	*n*	%
Demographic variables		
Gender		
Male	1447	31.0
Female	3220	68.9
Faculty		
Medicine	620	13.3
Arts and Education^a^	1198	25.6
Law	616	13.2
Management and Finance	1025	21.9
Science	687	14.7
School of Computing	524	11.2
Year of Study		
1^st^ year	1946	41.7
2^nd^ year	1243	26.6
3^rd^ year	838	17.9
4^th^ year	530	11.3
5^th^ year (Medicine)^b^	114	2.4
Age group (Mean = 22.17; *SD* = 1.46)		
18–20 years	515	11.0
21–23 years	3355	71.8
24 and above	793	17.0
Ethnicity		
Sinhala	4281	91.7
Tamil	193	4.1
Sri Lankan Moor	147	3.1
Other	46	1.0
Religion		
Buddhist	4064	87.0
Hindu	161	3.4
Islam	152	3.3
Roman Catholic	215	4.6
Other	73	1.6
Residence when going to University		
Home	1752	37.5
Hostel	1403	30.0
Rented place	1188	25.4
Home of friend or relative	272	5.8
Other	51	1.1
Other variables		
Exposure to problem through family/friends		
No	1773	38.0
Yes	1695	36.3
Don’t know	1054	22.6
Personal experience of problem		
No	2525	54.1
Yes	1511	32.3
Don’t know	326	7.0
If problem personally experienced (responding as ‘Yes’ or ‘Don’t know), if help sought (*n* = 1767)		
Help not sought	683	38.7
Tried first but got help later	704	39.8
Help sought	380	21.5
Screening positive for Major Depression (*n* = 4304)		
No	3903	90.7
Yes	401	9.3

^a^Those in the Faculty of Education were 5.6 % of this group

^b^Only those from the Faculty of Medicine had a 5^th^ year of study

Instances that the sum of participants do not equal the total number of respondents are due to missing data

The responses of 4559 participants met the criteria for being assessed using the Treatment Beliefs Scale (≤2 missing responses; responses of 112 participants excluded). The following internal consistency estimates were obtained for the scale: Cronbach’s alpha = 0.72, 95 % *CI* [0.71, 0.74]; McDonald’s Omega = 0.66, 95 % *CI* [0.64, 0.69]. The undergraduates obtained a mean score of 17.50 on this scale (95 % *CI* [17.41, 17.60]; *SD* = 3.34; Median = 18; Min = 0, Max = 23; mean score as a percentage = 76 %).

### Correlates of problem-recognition

A total of 4535 responses were obtained for the question relating to problem-recognition (136 missing responses). Table 2 presents the odds of problem-recognition in relation to the examined predictor variables when the analyses were both adjusted and unadjusted for the other variables. Only the adjusted odds ratios are discussed as they are indicative of the effects of the predictor variables on problem-recognition while taking into account the effects of other variables. As seen in Table 2, the odds of recognising the problem as depression was higher among females, those in the Medical Faculty and those in higher years of study (as compared to those in the first year). The odds of recognising depression in reference to the Medical Faculty varied across the different faculties. There were lower odds of recognition among those living in hostels (compared to home) and among Hindus (compared to Buddhists).

**Table 2:** Correlates/predictors of problem-recognition examined using multinomial logistic regression

		Depression				Other mental health-related problems			
	predictor variables	% recognising condition in relation to demographic subgroup	Unadjusted Odds Ratio	Adjusted Odds Ratio		% recognising condition in relation to demographic subgroup	Unadjusted Odds Ratio	Adjusted Odds Ratio	
			(*n* = 4189–4535)	[99 % CI]			(*n* = 4189–4535)	[99 % CI]	
				(*n* = 4171)				(*n* = 4171)	
**Gender:**	**Male**	19.2				46.0			
	Female	16.7	1.16	1.72^***^	[1.26, 2.34]	57.2	1.65^***^	1.32^**^	[1.06, 1.64]
**Faculty:**	**Medicine**	61.3				24.5			
	Arts and Education	4.2	0.04^***^	0.04^***^	[0.03, 0.08]	69.1	1.49^**^	1.32	[0.85, 2.03]
	Law	10.2	0.12^***^	0.12^***^	[0.07, 0.20]	69.3	1.95^***^	1.76^**^	[1.09, 2.84]
	Management and Finance	7.8	0.05^***^	0.04^***^	[0.03, 0.08]	55.0	0.85	0.83	[0.54, 1.28]
	Science	25.2	0.18^***^	0.20^***^	[0.12, 0.32]	42.9	0.77	0.75	[0.47, 1.20]
	Computer	13.7	0.08^***^	0.10^***^	[0.06, 0.16]	45.9	0.65	0.66	[0.41, 1.06]
**Year:**	**1** ^**st**^ **year**	10.5				58.2			
	2^nd^ Year	14.2	1.51^**^	1.76^***^	[1.19, 2.61]	57.7	1.11	1.13	[0.88, 1.45]
	3^rd^ Year	17.9	1.84^***^	1.66^**^	[1.07, 2.59]	53.0	0.98	0.91	[0.68,1.22]
	4^th^ Year	33.2	3.74^***^	3.03^***^	[1.74, 5.30]	40.3	0.82	0.84	[0.55, 1.28]
	5^th^ Year (Medicine)	91.9	101.48^***^	29.38^***^	[4.14, 208.58]	5.4	1.08	1.58	[0.17, 14.58]
**Age group:**	**18–20 years**	12.6				55.8			
	21–23 years	13.6	1.16	0.73	[0.44, 1.21]	57.1	1.10	1.25	[0.91, 1.73]
	24 and above	36.6	3.64^***^	0.74	[0.37,1.47]	38.2	0.86	1.17	[0.73, 1.89]
**Residence:**	**Home**	21.2				47.1			
	Hostel	15.2	0.82	0.44^***^	[0.30, 0.64]	57.2	1.39^***^	1.02	[0.79, 1.32]
	Rented place	15.0	0.85	0.77	[0.54, 1.12]	58.8	1.51^***^	1.29	[1.00, 1.66]
	Home of friend/relative	10.5	0.54^**^	0.69	[0.35, 1.34]	60.5	1.40	1.18	[0.78, 1.80]
	Other	46.0	3.44^**^	0.64	[0.16, 2.62]	34.0	1.14	1.02	[0.35, 2.98]
**Religion:**	**Buddhist**	16.8				55.5			
	Hindu	12.9	0.45^**^	0.20^***^	[0.09, 0.47]	40.1	0.43^***^	0.46^***^	[0.28, 0.76]
	Islam	18.9	0.84	1.33	[0.64, 2.73]	43.9	0.59^**^	0.57^**^	[0.33, 0.98]
	Roman Catholic	26.1	1.35	1.42	[0.79, 2.52]	42.2	0.66	0.70	[0.44, 1.11]
	Other	31.0	1.72	1.72	[0.68, 4.37]	39.4	0.66	0.74	[0.32, 1.67]
**Screening positive for Major Depression:**	**No**	18.1				54.1			
	Yes	15.9	0.73	0.76	[0.46, 1.24]	50.6	0.78	0.73	[0.53, 1.00]
**Nagelkerke R Square**					**0.30**				**0.30**

^**^ *p* < .01; ^***^ *p* < .001

Predictor variables in bold text indicate the demographic subgroups that were the reference groups for the dummy coded variables

The odds of using a mental health-related label to recognise the problem was higher among females and Law students (compared to Medical students). Lower odds of recognition were seen among those of the Hindu and Islam faiths (compared to Buddhists).

### Correlates of treatment beliefs

Table 3 presents the associations that each of the predictor variables had with scores on the Depression Treatment Beliefs Scale (as standardised regression coefficients), when adjusting and not adjusting for the other variables. Only the adjusted regression coefficients are discussed. Higher scores on the Depression Treatment Beliefs Scale were associated with being female, being in the Medical Faculty (compared to other faculties), being Roman Catholic (compared to Buddhist), seeking help for the problem after trying to deal with it alone (compared to not getting help) and recognising the problem as depression or recognising it using other mental health-related labels. Lower scores on this scale were associated with being in years 2–4 (compared to the first year), having family/friends with the described problem and having higher scores on the Social Distance Scale.

**Table 3:** Correlates/predictors of scores on Depression Treatment Beliefs Scale examined using linear regression

	Predictor variables	Subgroup score		Standardised regression coefficient (unadjusted)	Standardised regression coefficient	
					(adjusted) [99 % CI]	
		M (SD)		(*n* = 4233-4559)	(*n* = 3793)	
**Gender:**	**Male**	16.90	(3.52)			
	Female	17.78	(3.20)	0.12^***^	0.10^***^	[0.06, 0.15]
**Faculty:**	**Medicine**	18.30	(3.40)			
	Arts and Education	17.79	(3.13)	−0.07^**^	−0.11^***^	[−0.18, −0.04]
	Law	17.77	(3.14)	−0.05^**^	−0.11^***^	[−0.17, −0.05]
	Management and Finance	16.91	(3.40)	−0.17^***^	−0.18^***^	[−0.25, −0.11]
	Science	17.35	(3.30)	−0.10^***^	−0.11^***^	[−0.17, −0.05]
	Computer	16.96	(3.56)	−0.13^***^	−0.11^***^	[−0.17, −0.05]
**Year:**	**1** ^**st**^ **Year**	17.85	(3.09)			
	2^nd^ Year	17.10	(3.51)	−0.10^***^	−0.09^***^	[−0.14, −0.04]
	3^rd^ Year	17.24	(3.35)	−0.07^***^	−0.07^***^	[−0.12, −0.02]
	4^th^ Year	17.39	(3.47)	−0.04^**^	−0.06^**^	[−0.12, −0.01]
	5^th^ Year (Medicine)	18.55	(3.92)	0.03	−0.02	[−0.07, 0.03]
**Age group:**	**18–20 years**	17.83	(3.12)			
	21–23 years	17.47	(3.33)	−0.05	0.02	[−0.05, 0.08]
	24 and above	17.46	(3.46)	−0.04	0.03	[−0.05, 0.10]
**Residence**	**Home**	17.36	(3.46)			
	Hostel	17.60	(3.32)	0.03	−0.02	[−0.06, 0.03]
	Rented place	17.69	(3.17)	0.04	−0.01	[−0.06, 0.03]
	Home of friend/relative	17.20	(3.05)	−0.01	−0.02	[−0.06, 0.02]
	Other	17.23	(4.01)	−0.004	−0.04	[−0.08, 0.01]
**Religion**	**Buddhist**	17.45	(3.35)			
	Hindu	17.90	(3.14)	0.02	0.03	[−0.01, 0.07]
	Islam	17.83	(3.31)	0.02	0.01	[−0.03, 0.05]
	Roman Catholic	17.90	(3.17)	0.03	0.05^**^	[0.01, 0.09]
	Other	17.23	(3.44)	−0.01	−0.01	[−0.05, 0.03]
**Exposure to problem through family/friends**	**No**	17.98	(3.10)			
	Response: Yes	17.27	(3.44)	−0.10^***^	−0.06^**^	[−0.11, −0.01]
	Response: Don’t know	17.24	(3.32)	−0.09^***^	−0.04	[−0.09, 0.002]
**Personal experience of problem**	**No**	17.79	(3.21)			
	Response: Yes	17.16	(3.44)	−0.09^***^	−0.09	[−0.26, 0.08]
	Response: Don’t know	16.95	(3.27)	−0.07^***^	−0.06	[−0.14, 0.03]
**If personally experienced, if help sought**	**Help not sought**	16.67	(3.53)			
	Tried first but got help later	17.52	(3.22)	0.09^***^	0.07^***^	[0.02, 0.12]
	Help sought	17.22	(3.43)	0.04	0.04	[−0.01, 0.08]
	Not relevant	17.04	(3.56)	0.16^***^	0.01	[−0.17, 0.19]
**Screening positive for Major Depression**	**No**	17.62	(3.26)			
	Yes	16.76	(3.85)	−0.08^***^	−0.04	[−0.08, 0.002]
**Recognition of problem**	**Not recognised**	16.77	(3.51)			
	Recognised as ‘depression’	18.32	(3.21)	0.18^***^	0.14^***^	[0.08, 0.19]
	Recognised using other mental health-related labels	17.70	(3.15)	0.14^***^	0.10^***^	[0.06, 0.15]
**Stigma scale scores**	Weak-not-Sick scale score	*NA*	*NA*	−0.04^**^	−0.04	[−0.08, 0.00]
	Dangerous-Undesirable scale score	*NA*	*NA*	−0.10^***^	−0.04	[−0.08, 0.003]
	Social Distance scale score	*NA*	*NA*	−0.16^***^	−0.16^***^	[−0.20, −0.12]
**Adjusted R square**					**.11**	

^**^ *p* < .01; ^***^ *p* < .001

Predictor variables in bold text indicate the demographic subgroups that were the reference groups for the dummy coded variables

## Discussion

This study examined the depression literacy of undergraduates in Sri Lanka, in relation to their ability to recognise the problem and their related treatment beliefs, and the correlates of their depression literacy. Although their ability to recognise depression was low, they had 76 % agreement with mental health experts about ways of dealing with depression, with this rate being similar to that obtained in the Reavley et al. [29] study. The findings also show that factors such as gender, discipline, year of study, exposure to depression and stigma are correlates of their depression literacy. Furthermore, their ability to recognise the problem was associated with better treatment beliefs.

Findings that female undergraduates have better depression literacy than their male counterparts, given their higher recognition of the problem specifically as depression or less-specifically by using other mental health-related labels as well as their higher agreement with mental health experts about ways of dealing with depression, align with previous problem-recognition studies among undergraduates [31, 42], and those that have generated mental health literacy scores of the general population [27, 29]. While such findings might be reflecting actual mental health literacy deficits among males, they might be also related to their characteristics of masculinity and reluctance to acknowledge mental illness among themselves [43] or seek assistance for it [44].

As would be expected, when compared to those in other faculties, medical undergraduates were better at recognising the condition as depression and showed greater alignment with expert opinion about treatments and options of help for depression . This concurs with previous findings, that undergraduates with more opportunities for exposure to mental health information have better literacy related to these issues [42, 45]. A related expectation might be that undergraduates in higher years of study, who are more likely to be exposed to such health information, to have higher levels of depression literacy. Although this expectation is supported by our finding that recognition of depression is better among those in senior years as compared to the first-years, it is contradicted by the finding that it is those in the first year who show greater agreement with expert opinion about treatments and options of help for depression. Interestingly, although 5^th^ year medical undergraduates exhibited greater ability to recognise depression as compared to first-years, the two groups did not differ in relation to their treatment belief scores. Hence, it is necessary to examine whether more years of study are associated with scepticism about the recommended treatments and options of help for depression, and importantly the factors affecting the treatment beliefs of 5^th^ year medical undergraduates who are at the culmination of their undergraduate medical training and expected to possess greater knowledge about appropriate help.

It is noteworthy that those who had been exposed to the problem through family and friends also had lower scores on the Depression Treatment Beliefs Scale indicating their lower depression literacy in relation to their treatment beliefs. This deviates from previous findings in Australia that show instead, that those with such exposure exhibit higher mental health literacy [29]. Such findings are especially concerning in relation to Sri Lanka as there might be a need for those close to mental health sufferers to be knowledgeable about help appropriate for these persons, as they might have to take pivotal roles in their care given the limited professional mental health services in the country. The regression coefficients presented in Table 3 also indicate a consistent pattern of lower scores on the Depression Treatment Beliefs Scale among those exposed to depression; i.e., those exhibiting symptomatology of a diagnosis, those reporting past experiences of the problem in oneself and through one’s social network. The findings point towards examining if in this population, the phenomenology of depression is related to pessimistic expectations about treatments and options of help for depression. The Sri Lankan mental health literacy survey of carers of patients with mental illness, done among those attending community clinics at a National Hospital, found that despite their high endorsement of professional mental health services, almost a quarter of respondents endorsed the option of dealing with the problem alone [18]. This further indicates the need to examine the reasons for such responses.

It is necessary to consider whether such findings are indicating *actual* negative experiences of these groups with the recommended options of help. However, the pattern of higher scores on the Depression Treatment Beliefs Scale among those who had experienced the problem and sought help for it as compared to those not seeking help, suggest that such individuals’ potential interactions with these options of help were positive. Therefore, while there might be other factors affecting the appraisals of these groups about these options, the findings indicate that the act of reaching out for help might positively affect such appraisals. This emphasises the need to facilitate interactions between these affected groups and the recommended options of help.

The findings also indicate that those with a greater need for social distance from sufferers have lesser agreement with mental health experts about ways to deal with depression. While these findings are in line with previous research on the influence of stigma on help-seeking beliefs [26], they also provide caution, as such beliefs and stigma held by the undergraduates could influence their use of appropriate treatments for their problems [46, 47]. However, it is encouraging that this undergraduate population had low stigmatising attitudes in relation to the Social Distance Scale [25].

There was a positive association between the two aspects of depression literacy that were examined. Recognising the problem as depression as well using other mental health-related labels for this purpose, were associated with more appropriate treatment beliefs as per expert consensus, which in turn, are expected to trigger related actions [48]. However, there was not always consistency in the predictors of these two aspects of depression literacy, as seen in the case of year of study, indicating that depression literacy is a complex construct.

The findings indicate that there are differences in these aspects of depression literacy among the different segments of this undergraduate population and that this might be associated with variations in their individual characteristics, experiences and attitudes about depression and help-seeking. However, it must be noted that the effect sizes of some of these examined relationships were small and that the clinical significance of these findings might also be small. Nevertheless, as some of the findings, such as the pattern of lower scores on the Depression Treatment Beliefs Scale among those exposed to depression, deviates from trends that might have been expected as per previous research, further examination of this construct both among undergraduates and the general population is recommended.

The current findings offer a few general recommendations for the design of depression literacy initiatives for this undergraduate population, but must also be verified in future research. The list of recommended options of help obtained through expert consensus provides guidance for developing services appropriate for undergraduates in the Sri Lankan context and also indicates the support networks which need to be strengthened and educated to provide the necessary mental health assistance to distressed undergraduates. Findings that approximately one tenth of these undergraduates are at risk of depression [24] stress the need to urgently address their low rates of depression recognition. This is further emphasised by our findings that the ability to recognise the problem is associated with higher agreement with experts about ways to deal with depression. Hence, educating this population to recognise the condition could be expected to trigger appropriate treatment beliefs and recommended help-seeking behaviours. However, depression literacy initiatives must address both the population’s ability to recognise the problem and their treatment beliefs simultaneously as their related educational needs might be different in relation to certain population factors (e.g., in the case of year of study). Furthermore, given that the findings indicate that there are various population factors that are associated with differences in these aspects of depression literacy, it is also necessary to consider that there might be different educational needs in the population (e.g. although the treatment beliefs of both males and those exposed to depression might need improvement the factors affecting their treatment beliefs might be different). Hence, these depression literacy initiatives cannot be only limited to educating individuals about depression and seeking help for it, but need to also address the unique constellation of factors that might affect their knowledge and perceptions about depression and interfere with their help-seeking. Therefore, such educational initiatives cannot be a “one-size-fits-all” package and if implementing generic programmes, these need to be supplemented by efforts focussing on the specific educational needs of the target audiences. Furthermore, such initiatives must also address other factors such as stigma, which might negatively impact beliefs and practices relating to the different options of help.

The findings need to be considered in light of the limitations of the study. In real life, the situation described in the vignette might present a complex interplay of factors that the vignette methodology might not have captured adequately. Furthermore, the cross-sectional design does not permit for any causal interpretations of the examined variables. Although the Cronbach’s Alpha of the constructed scale provides some indication that it is suitable for measuring the related construct in the population, the McDonald’s Omega that was obtained was comparatively lower. Hence, further examination of the psychometric properties of the measure is recommended. This study created a scale for assessing beliefs about treatments and options of help for dealing with depression using the views of mental health professionals as the validity standard. Future work could usefully explore the factor structure of undergraduates' beliefs to see if separate factors for various health belief systems emerge, as found in previous studies [49, 50]. Although the lower limit of the range of scores, which was zero, might indicate that those obtaining this score (n = 2) were not in agreement with experts about the helpfulness of all 23 scale items, such low scores might also be due to their uncertainty about the helpfulness of these options (by selecting the rating options “neither helpful nor unhelpful” or “don’t know”) or the poor validity of their responses. Hence further development of the scale to assess subtleties in participant responses and incorporation of items to assess the validity of the responses would facilitate the interpretation of findings in future work. As there was some indication that exposure to depression might be associated with lesser agreement with experts about the ways to deal with depression, further examination of this finding in relation to the nature of this exposure, such as its duration and the respondent’s relationship with the affected person, is also recommended.

The large sample size and the high response rate in the study reduce the likelihood of bias in the sample. Although this study was only conducted in one University in Sri Lanka, the large sample size, including undergraduates from diverse disciplines and all years of study, and its reflection of the demographic composition of the undergraduate population in Sri Lanka [30], also indicates that the findings provide an useful estimate of depression literacy among undergraduates in Sri Lanka.

## Conclusions

The undergraduates showed agreement with expert opinion about treatments and options of help for depression indicating their depression literacy in this regard. Recognition of the problem as depression or the use of a mental health-related label for this purpose was associated with greater alignment with such expert opinion. However, in the case of year of study, although recognition of depression was higher among those in higher years, they showed lesser agreement with experts about ways of dealing with the problem. Ability to recognise the problem as depression was lower among males and those from non-medical disciplines. They also showed lesser agreement with experts about treatments and options of help for depression. The latter was also seen among those exposed to the problem through their family and those desiring social distance from their peers with depression.

## Additional file

Additional file 1:
**English-Sinhala version of questionnaire.** (PDF 189 kb)

## Abbreviations

DV: Dependent variable
IV: Independent variable
PHQ-9: Patient Health Questionnaire-9
